# *In silico* identification of genetic mutations conferring resistance to acetohydroxyacid synthase inhibitors: A case study of *Kochia scoparia*

**DOI:** 10.1371/journal.pone.0216116

**Published:** 2019-05-07

**Authors:** Yan Li, Michael D. Netherland, Chaoyang Zhang, Huixiao Hong, Ping Gong

**Affiliations:** 1 Bennett Aerospace, Inc., Cary, North Carolina, United States of America; 2 Environmental Laboratory, U.S. Army Engineer Research and Development Center, Vicksburg, Mississippi, United States of America; 3 School of Computing Sciences and Computer Engineering, University of Southern Mississippi, Hattiesburg, Mississippi, United States of America; 4 Division of Bioinformatics and Biostatistics, National Center for Toxicological Research, U.S. Food and Drug Administration, Jefferson, Arkansas, United States of America; Nanjing University, CHINA

## Abstract

Mutations that confer herbicide resistance are a primary concern for herbicide-based chemical control of invasive plants and are often under-characterized structurally and functionally. As the outcome of selection pressure, resistance mutations usually result from repeated long-term applications of herbicides with the same mode of action and are discovered through extensive field trials. Here we used acetohydroxyacid synthase (AHAS) of *Kochia scoparia* (*Ks*AHAS) as an example to demonstrate that, given the sequence of a target protein, the impact of genetic mutations on ligand binding could be evaluated and resistance mutations could be identified using a biophysics-based computational approach. Briefly, the 3D structures of wild-type (WT) and mutated *Ks*AHAS-herbicide complexes were constructed by homology modeling, docking and molecular dynamics simulation. The resistance profile of two AHAS-inhibiting herbicides, tribenuron methyl and thifensulfuron methyl, was obtained by estimating their binding affinity with 29 *Ks*AHAS (1 WT and 28 mutated) using 6 molecular mechanical (MM) and 18 hybrid quantum mechanical/molecular mechanical (QM/MM) methods in combination with three structure sampling strategies. By comparing predicted resistance with experimentally determined resistance in the 29 biotypes of *K*. *scoparia* field populations, we identified the best method (i.e., MM-PBSA with single structure) out of all tested methods for the herbicide-*Ks*AHAS system, which exhibited the highest accuracy (up to 100%) in discerning mutations conferring resistance or susceptibility to the two AHAS inhibitors. Our results suggest that the *in silico* approach has the potential to be widely adopted for assessing mutation-endowed herbicide resistance on a case-by-case basis.

## Introduction

Acetohydroxyacid synthase (AHAS, also known as acetolactate synthase or ALS) is a group of biosynthetic enzymes found in all plants, fungi, and bacteria (but absent in animals and humans). AHAS is a key enzyme that catalyzes the formation of acetolactate and acetohydroxybutyrate from pyruvate and 2-ketobutyrate [1, 2]. This is the first step in biosynthesis of the essential branched-chain amino acids (valine, leucine, and isoleucine), which are critical for all forms of life. AHAS has long been an attractive target in the development of herbicides, fungicides, and antimicrobials because its inhibitors have a low toxicity to mammals while still being highly selective and very potent [3]. AHAS-inhibiting herbicides are the largest site-of-action group on the market, with more than 50 chemicals belonging to five classes (sulfonylaminocarbonyltriazolinones, triazolopyrimidines, pyrimidinyl(thio)benzoate, sulfonylureas, and imidazolinones) and sulfonylureas being the majority [4]. However, persistent use of herbicides has exerted intense selection pressure on a great variety of weed species and resulted in the evolution of resistance [5]. In the most common mechanism, resistance is conferred by alteration of amino acids in the target site that attenuates the sensitivity to target-specific herbicides [6, 7]. The magnitude of herbicide resistance depends on weed species, structural change induced by mutation, and the type of herbicide. For a specific herbicide, a given mutation may endow moderate to high resistance [7, 8] or, in rare instances, an increase in sensitivity to the herbicide in different species [5]. In the current practice of weed control, resistance mutations may be discovered only after repeated failure of herbicide application. Therefore, there is a strong and urgent demand for a reliable and systematic approach for determining resistance profiles of different herbicides that are in use or have been newly developed before commencing weed treatment. Compared to wet lab-based experiments and techniques, computational approaches provide a rapid and cost-effective solution to screen and detect resistance mutations.

Although computational endeavors in understanding herbicide resistance have been scarcely reported [8, 9], considerable *in silico* efforts have been made to interpret and predict drug resistance associated with genetic mutations during the last decade [10–14]. Here we focus on computational studies in which the mutational effect is evaluated by measuring protein-ligand interactions. A handful of biophysics-based methods have been employed to estimate the affinity of inhibitors binding to wild-type (WT) or mutated proteins [15–22], and the results are in good agreement with experimental data. Moreover, viable mutations that confer resistance to an inhibitor of dihydrofolate reductase have been predicted by a protein design algorithm before being verified by crystallography and other experiments [23]. In addition to mutational effects on binding affinity, the influence of mutations on catalytic activity has been studied [24]. A successful resistance mutation should only impede the inhibitor binding to the enzyme, but not the catalytic efficacy. In the aforementioned reports, the noncovalent interaction between protein and ligand is typically described by a molecular mechanics (MM) potential function. Despite the success of MM force fields, it is always of immense interest to precisely treat noncovalent interactions for accurate calculation of binding affinity. In theory, noncovalent interactions can be handled more accurately with quantum mechanics (QM) than with MM [25, 26] because important effects such as charge transfer and electronic polarization are considered in QM, but not in MM. Inevitably, these additional considerations cause a drastic increase in computational requirements, which limits the size of the systems that can be studied. As a tradeoff between efficiency and accuracy, semi-empirical quantum mechanical (SQM) methods have lately attracted attention again [27, 28]. SQM-DH, an improved SQM approach with the addition of dispersion (D) and hydrogen bond (H) correction terms, yields results comparable to high-level QM calculations in terms of accuracy [28, 29]. Even so, SQM is still too computationally demanding to be applicable for a large biomacromolecular system. A feasible solution is the hybrid QM/MM model, in which the interaction region is treated by QM whereas the remaining part is described by MM. Consequently, a number of QM/MM approaches have been developed to study a group of ligands binding to a receptor [25]. However, it is unknown how well these QM/MM methods could differentiate between mutations that confer resistance or susceptibility to an herbicide.

Here we investigate if the MM and QM/MM approaches can correctly identify AHAS mutations in *Kochia scoparia* (also called *Bassia scoparia*) that confer resistance to two sulfonylurea herbicides, tribenuron methyl and thifensulfuron methyl. *K*. *scoparia* is one of the most problematic annual broadleaf weeds in North America known for its rapid adaptability and widespread herbicide resistance [30]. So far, it has been reported that resistance to herbicides arises via multiple mechanisms of action [31]. The resistance mechanism of *K*. *scoparia* to AHAS-inhibiting herbicides, including the two most popular classes of AHAS inhibitors (sulfonylurea and imidazolinone), has been well studied. Principally, this resistance is acquired through mutations in the *AHAS* gene, which inhibit herbicide binding, but do not severely impair AHAS catalytic activity and plant growth [30, 32, 33].

Because the structure of *K*. *scoparia* AHAS (*Ks*AHAS) has not been solved, we first modeled the structures of WT and mutated *Ks*AHAS in complex with the two sulfonylurea herbicides using homology modeling and docking. Then a plethora of MM and QM/MM methods were employed to calculate the binding affinity, including MM-PBSA/GBSA/ALPB (i.e., MM combined with Poisson-Boltzmann/generalized Born/Analytical Linearized Poisson-Boltzmann and surface area continuum solvation) and QM/MM-GBSA combined with SQM and SQM-DH approaches. The estimation of binding affinity was based on single structure or an ensemble of structures sampled from classical and QM/MM molecular dynamics (MD) simulations.

## Materials and methods

### Data curation and structure preparation

**Fig 1:**
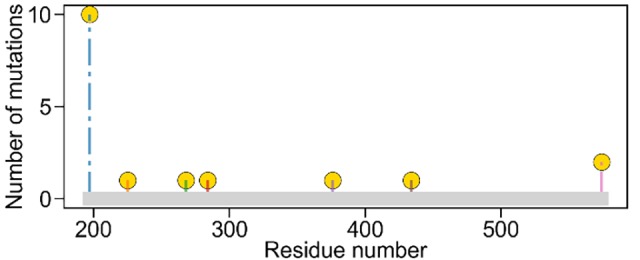
Reported mutation sites in *Kochia scoparia* AHAS (*Ks*AHAS). Mutations at four sites (Pro197, Val225, Asp376, and Trp574) confer resistance to sulfonylurea herbicides while mutations at the other three sites (Gly268, Glu284, and Asn434) resulted in susceptibility. Residue numbers refer to the positions in the *Arabidopsis thaliana* AHAS (*At*AHAS) amino acid sequence with which *Ks*AHAS was aligned.

Various biotypes with AHAS mutations have been reported in field *K*. *scoparia* populations [31, 34–36]. We curated 28 amino acid substitutions (**Table 1**) occurring at 7 *Ks*AHAS residue sites (according to the *Arabidopsis thaliana* AHAS (*At*AHAS) amino acid sequence): Pro197, Val225, Gly268, Glu284, Asp376, Asn434, and Trp574 (**Fig 1**). These residues corresponded to Pro189, Val217, Gly260, Glu276, Asp372, Asn430, and Trp570 in *Ks*AHAS. In the remainder of this paper, residue numbers refer to those in *At*AHAS. Among the 28 *Ks*AHAS mutations, 25 (including 14 single-point and 11 double-point mutations that were all labeled “R”) endowed resistance to the two sulfonylurea herbicides, tribenuron methyl and thifensulfuron methyl, and the remaining 3 mutations (labeled “S”) caused susceptibility to them (**Table 1**). Out of the 14 single-point resistance mutations, 10 occurred at the site of Pro197 (**Fig 1** and **Table 1**).

**Table 1:** AHAS mutations in field *Kochia scoparia* populations along with their experimentally determined resistance to tribenuron methyl and thifensulfuron methyl.

**Mutation type**	**Residue substitution**		**Activity**	**Reference**
Wild type	None		S^a^	33,34
Single mutation	Pro197	Ala	R^b^	33
		Arg	R	33–35
		Gln	R	33–35
		Glu	R	30
		Leu	R	33
		Lys	R	33,34
		Met	R	33
		Ser	R	33,35
		Thr	R	33,34
		Trp	R	33
	Val225	Ile	R	33
	Gly268	Asp	S	33,34
	Glu284	Val	S	33,34
	Asp376	Glu	R	33–35
	Asn434	Lys	S	33
	Trp574	Leu	R	33–35
		Arg	R	33
Double mutation	Pro197Ala+Trp574Leu		R	34
	Pro197Gln+Asp376Glu		R	33
	Pro197Ser+Asp376Glu		R	33
	Pro197Thr+Asp376Glu		R	33
	Pro197Arg+Trp574Leu		R	33
	Pro197Gln+Trp574Arg		R	33
	Pro197Gln+Trp574Leu		R	33–35
	Pro197Leu+Trp574Leu		R	33
	Pro197Ser+Trp574Leu		R	33,34
	Pro197Thr+Trp574Leu		R	33,34
	Asp376Glu+Trp574Leu		R	33,35

^a^R = resistant

^b^S = susceptible

**Fig 2:**
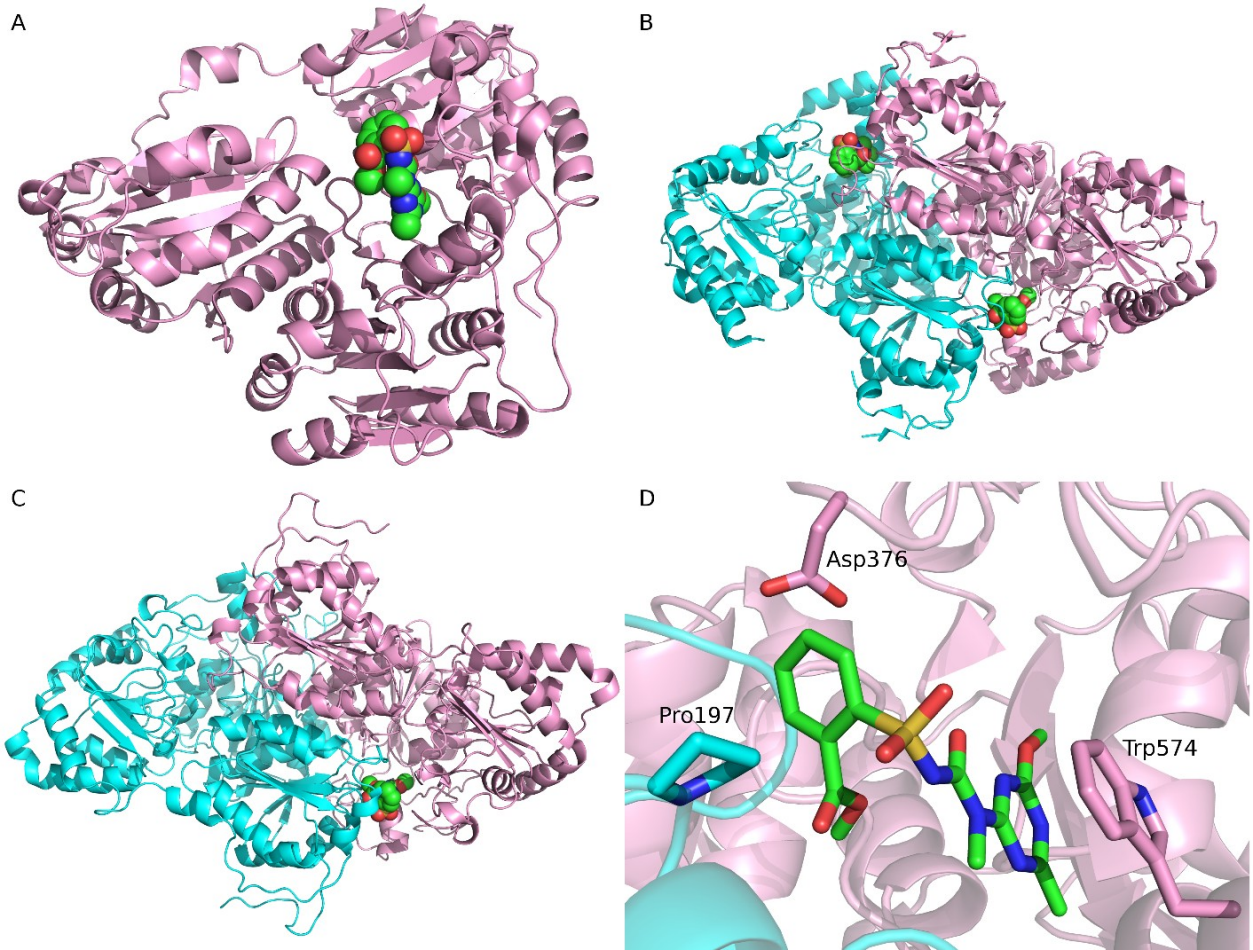
The complex structures of AHAS bound with tribenuron methyl. (A) The monomer structure of *Arabidopsis thaliana* AHAS (*At*AHAS) bound with tribenuron methyl (1YI1). (B) The dimer structure of *Saccharomyces cerevisiae* AHAS (*Sc*AHAS) bound with tribenuron methyl (1T9A). (C) The modeled dimer structure of *Kochia scoparia* AHAS (*Ks*AHAS) bound with tribenuron methyl. (D) Important interactions between *Sc*AHAS and tribenuron methyl (1T9A). In panels A, B, and C, tribenuron methyl is displayed in spheres, indicating the binding site. In panels B, C, and D, the two chains of AHAS are colored pink and cyan, respectively. In panel D, tribenuron methyl and three important residues are shown in sticks. Carbons in tribenuron methyl are colored green.

The structures of *At*AHAS and *Saccharomyces cerevisiae* AHAS (*Sc*AHAS) have been solved experimentally. All *At*AHAS structures are monomers, whereas dimer structures are available for *Sc*AHAS. The structures of *At*AHAS (PDB ID: 1YI1, monomer) [37] and *Sc*AHAS (PDB ID: 1T9A, dimer) [38], both bound with the herbicide of tribenuron methyl, were retrieved from the Protein Data Bank (**Fig 2**). The WT *K*. *scoparia* AHAS sequence (GI 188529573) [33] was downloaded from NCBI (https://www.ncbi.nlm.nih.gov/protein/188529573). The 3D structures of tribenuron methyl and thifensulfuron methyl were retrieved from PubChem. The two herbicides were prepared by adding hydrogen with Open Babel 2.4 [39] and assigning AM1-BCC atom charges with Antechamber implemented in Amber 16 [40].

### Molecular docking and homology modeling

*At*AHAS shared a sequence similarity of about 80% with *Ks*AHAS, whereas the sequence similarity between *Sc*AHAS and *Ks*AHAS was about 40%, which allowed us to build homology models. First, the dimer structure of 1YI1 (*At*AHAS) was modeled using the structure of 1T9A (*Sc*AHAS) as the template. Then, tribenuron methyl and thifensulfuron methyl were docked to the dimer structure of 1YI1 using DOCK 6.7 [41]. Finally, using the docked structures as templates, *Ks*AHAS structures bound with tribenuron methyl or thifensulfuron methyl were constructed using Modeller 9.17 [42]. For each herbicide, 29 *Ks*AHAS (1 WT and 28 mutated) structures were generated. In docking simulations, the receptor box delimiting the binding pocket was calculated with SHOWBOX. Potential grids were generated by the GRID program using a 0.3 Å spacing. The herbicides were flexibly docked into the *At*AHAS or *Ks*AHAS structure, and the number of sampled ligand poses was set to 1,000.

### Molecular dynamics (MD) simulations

One of the two symmetry binding sites in *Ks*AHAS was removed for reduction of computational complexity. The remaining protein with two chains and 484 residues was used for MD simulation and calculation of binding affinity. The WT and mutated *Ks*AHASs were modeled using the Amber ff14SB force field [43]. The herbicides were modeled using the GAFF2 force field [44]. Each complex structure was explicitly solvated in a rectangular box of TIP3P water molecules with a minimal distance of 10 Å from the protein to the box edges. Counter ions (Na+) were added to neutralize uncompensated charges. The whole system was energy minimized with 2,000 steps of steepest descent followed by 10,000 steps of conjugate gradient without any harmonic restraint. The energy-minimized structures were extracted from the water box for calculation of the single-point binding free energy.

Then, coupled to a Langevin thermostat, the system was heated from 10 K up to 300 K by increments of 100 K in 20 ps and continued to run for 40 ps at 300 K at constant volume. Finally, the system was equilibrated for 200 ps in NPT ensemble with the Langevin thermostat and isotropic position scaling at 300 K and 1 bar, respectively. The production run for each complex structure was carried out for 2 ns in NVT ensemble with the Langevin thermostat at 300 K using the parallel PMEMD. The MD simulations ran only for 2 ns because it was previously reported that the length of MD simulations had little effect on the calculation of binding affinity [45]. After that, the QM/MM [46, 47] MD simulations were turned on. The QM region was composed of the herbicide and side chains of residues at the 7 mutation sites except Gly268 and Pro197. Gly268 was excluded because it was located outside the QM region. For Pro197, only backbone atoms were included. Thus, the QM region contained 89–124 atoms, depending on herbicides and mutations. The AM1 method with dispersion correction (AM1D) was employed to model the QM region. The QM/MM MD simulations ran for 200 ps in NVT ensemble with the Langevin thermostat at 300K using the parallel SANDER. The trajectories were sampled at a time interval of 10 ps. In our case, the QM/MM MD simulations were 45–120 times slower than the classical MD simulations, depending on the size of the QM region. For each classical MD trajectory, the last 50 frames were obtained for calculation of binding free energy. For any QM/MM MD trajectory, the last 10 frames were extracted to calculate the binding free energy.

All MD simulations were carried out using Amber 16 [40]. The equations of motion were solved with the leapfrog integration algorithm with a time step of 2 fs. The lengths of all bonds involving hydrogen atoms were kept constrained with the SHAKE algorithm. The particle mesh Ewald (PME) method was applied for treating long-range electrostatic interactions. Periodic boundary condition was used in all simulations that were performed on a Cray XE6 High Performance Computing system with 32 CPUs on each node.

### Binding free energy calculation

In MM-PBSA [48], binding affinity (*ΔG*_*bind*_) was estimated from free energies of reactants (receptor and ligand) and product (complex): Δ*G*_*bind*_ = *G*_*complex*_−(*G*_*receptor*_+*G*_*ligand*_). The free energy of a state (receptor, ligand, or complex) was decomposed into gas-phase molecular mechanics energies (*E*_*MM*_), solvation energies (*G*_*solv*_), and conformational entropy (*TS*). The standard molecular mechanics energy included internal (bond, angle, and dihedral), electrostatic, and van der Waals interactions. The solvation energy was determined by the polar (*G*_*pol*_) and nonpolar (*G*_*np*_) contributions. The polar solvation contribution was calculated by solving the Poisson–Boltzmann (PB) equation, and the nonpolar contribution was estimated by the solvent accessible surface area (SASA). The entropy contribution was obtained by normal mode analysis or quasi-harmonic approximation, which was dropped in our calculation. Thus, the binding affinity was estimated as the sum of the three energy terms (Eqs 1 and 2).

$$G=E_{MM}+G_{solv}-TS=E_{inter}+E_{ele}+E_{vdW}+G_{pol}+G_{np}-TS$$ (1)

$$\Delta G_{bind}=G_{complex}-(G_{receptor}+G_{ligand})=\Delta E_{MM}+\Delta G_{solv}-T\Delta S$$ (2)

The polar solvation contribution (*G*_*pol*_) was also estimated using the generalized Born (GB) method, a computationally efficient approximation to the PB equation. In this work, two GB models (the improved GB^OBC^ model [49, 50] and the GBn model [51]) were tested. ALPB (Analytical Linearized Poisson-Boltzmann) [52, 53] was another approximation method we tested to handle electrostatic interactions within the implicit solvent model.

In QM/MM-GBSA [46, 54], the mechanics energies (*E*_*MM*_) were replaced with the QM/MM energies (Eq 3).

$$\Delta G_{bind}=\Delta (E_{QM}+E_{MM}+E_{QM/MM})+\Delta G_{solv}-T\Delta S$$ (3)

A series of SQM methods were used for the QM calculation, including PM3 [55], RM1 [56], DFTB3 [57], AM1, PM6, and AM1/PM6 coupled with the empirical dispersion (D) and hydrogen bonding (H) correction [58] (i.e., AM1D, AM1DH, PM6D, and PM6DH). For simplicity, a QM/MM-GBSA method is represented by the SQM method plus the GB model in the remaining text. For example, AM1D-GBn stands for QM/MM-GBSA combined with AM1, dispersion correction and the GBn model.

The calculations of binding free energy were performed with SANDER implemented in Amber 16. In MM-PBSA, the internal dielectric constant (ε) was set to 2 and 4, as previously described [45]. Other parameters were kept at their default settings. The binding free energy was calculated using single structure as well as ensembles of conformations obtained from classical and QM/MM MD simulations.

### Prediction power assessment

For each combination of herbicide and binding free energy estimation method, the 29 *Ks*AHASs were sorted by the binding free energy in descending order (i.e., from weak binding to strong binding), and the top-ranked *Ks*AHASs were predicted to be potentially resistant. Based on the known field resistance data, we made such prediction calls as true resistance (TR) and false resistance (FR) in the top *n Ks*AHASs, and true susceptibility (TS) and false susceptibility (FS) in the remaining *(29 –n) Ks*AHASs. Out of the 29 *Ks*AHASs, 25 contained resistance mutations, which should ideally be ranked ahead of the other 4 *Ks*AHASs (3 mutated plus 1 WT). If a resistant *Ks*AHAS was ranked below 25, it was called a FS; if a susceptible *Ks*AHAS was found in the top 25, it was called a FR. The following three metrics were introduced to compare the overall prediction power of the methods for binding affinity calculation: accuracy, enrichment factor (EF), and AUC-ROC (see below for definition and explanation).

Accuracy (Ac) was defined as $\frac{TR+TS}{N}$, where *N* was the total number of AHASs.

EF was defined as $\frac{a/n}{A/N}$ in the top-ranked 10 AHASs, where *N* was the total number of AHASs; *n* was the number of AHASs selected (that is, 10); *A* was the total number of resistant AHASs; and *a* was the number of resistant AHASs in the selection.

The receiver operating characteristic (ROC) [59] curve was drawn by plotting the TR rate ($\frac{TR}{TR+FS}$) against the FR rate ($\frac{FR}{FR+TS}$) with an increasing *n*. Here, the area under the curve (AUC) of ROC was the integral of the ROC plot, and served as a measure of the probability that a binding free energy estimation method could correctly rank a resistant AHAS over a susceptible AHAS.

The paired *t*-test hypothesis testing method was employed to assess if two groups of data were statistically different from each other. The null hypothesis was that there was no significant difference between the two groups. The paired *t*-test is a parametric statistic with an assumption that the data follow a normal distribution. All hypothesis testing was carried out at the 5% significance level using Python 3.4 and SciPy 0.18.

## Results

### Modeling of *Ks*AHAS-herbicide structures

Crystallographic studies indicate that AHAS inhibitors bind in a channel leading to the active site on the interface of the AHAS dimer and consequently block substrate access to the catalytic site [37, 38]. While the complex structures of tribenuron methyl with *At*AHAS and *Sc*AHAS have been experimentally determined (**Fig 2A and 2B**), the structure of AHAS-thifensulfuron methyl was unavailable at the time of this study. Using the known structures as templates, the *Ks*AHAS-herbicide complexes were built using homology modeling as described in the **Materials and methods** section (**Fig 2C**). Inspection of the binding mode of *Sc*AHAS and tribenuron methyl (PDB entry 1T9A) showed that Trp574 formed the π-π interaction with the heterocyclic ring of tribenuron methyl while Pro197 and Asp376 were in close contact with the aromatic ring (**Fig 2D**). The three residues (Pro197, Asp376, and Trp574) are highly conserved in *AHAS* genes [6], and the aforementioned interactions can be found in other AHAS-herbicide complexes as well as in the modeled *Ks*AHAS-herbicide structures. Mutations at these conserved residue sites may disrupt the important interactions between AHAS and herbicides (thereby disturbing herbicide binding), which may explain why resistance mutations mostly occurred at these three sites (see **Fig 1** and **Table 1)**.

**Fig 3:**
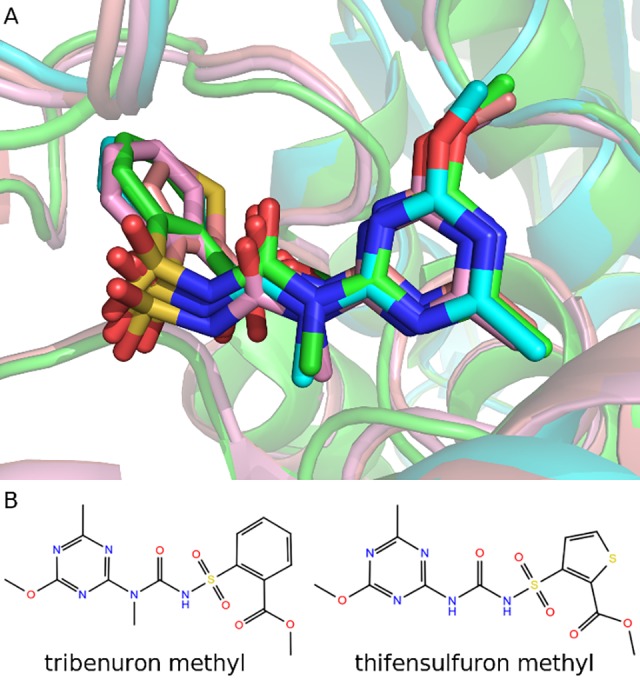
**Comparison of the two sulfonylurea herbicides binding to *At*AHAS, *Sc*AHAS, and *Ks*AHAS (A) and chemical structures of tribenuron methyl and thifensulfuron methyl (B).** *At*AHAS and *Ks*AHAS are superposed onto *Sc*AHAS. Carbons in *At*AHAS-tribenuron methyl (1YI1), *Sc*AHAS-tribenuron methyl (1T9A), *Ks*AHAS-tribenuron methyl, and *Ks*AHAS-thifensulfuron methyl complexes are colored cyan, green, pink, and salmon, respectively.

Examination of AHAS-herbicide crystal structures suggests that the same herbicide adopts nearly identical orientation when bound to AHASs in different species. For example, the poses of tribenuron methyl in complex with *At*AHAS and *Sc*AHAS were very much alike with a ligand RMSD (root mean square deviation) of 0.79 Å (**Fig 3A**). In the modeled *Ks*AHAS-tribenuron methyl complex, the ligand pose was almost the same as those experimentally determined with a ligand RMSD of 0.67 Å (*At*AHAS) or 1.07 Å (*Sc*AHAS). In the *Ks*AHAS-thifensulfuron methyl complex, the ligand exhibited a binding mode highly similar to that of tribenuron methyl in AHAS. As shown in **Fig 3A**, the central sulfonylurea bridge and heterocyclic ring of the herbicides completely overlapped, while the thiophene ring of thifensulfuron methyl moved slightly away from the aromatic ring of tribenuron methyl. This was possibly because chemical structures of the two herbicides were very similar with the aromatic ring in tribenuron methyl substituted by the thiophene ring in thifensulfuron methyl (**Fig 3B**), which induced the rearrangement.

### Prediction performance comparison

**Fig 4:**
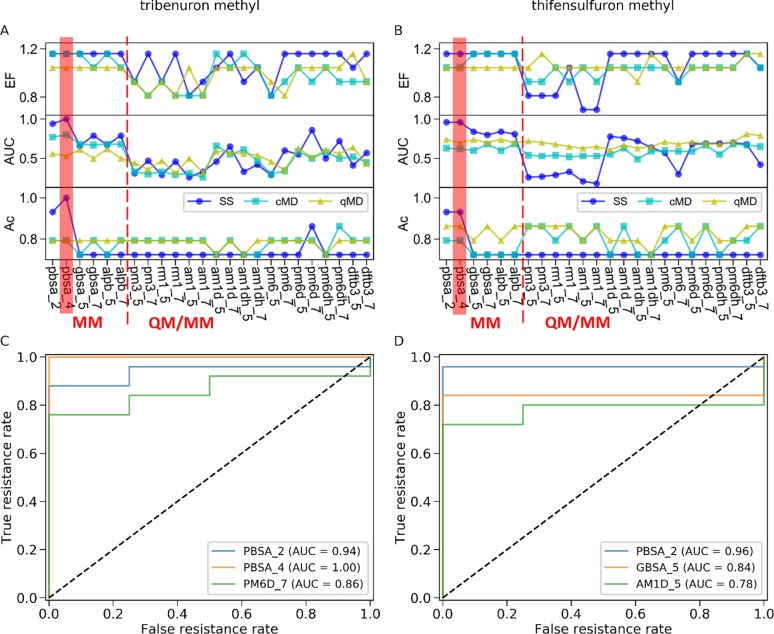
Prediction performance of 24 binding affinity estimation methods in combination with three conformational sampling strategies evaluated by EF, AUC, and accuracy (Ac). (A) Performance metrics for prediction on resistance to tribenuron methyl. (B) Performance metrics for prediction of resistance to thifensulfuron methyl. (C) The top three ROCs for tribenuron methyl. (D) The top three ROCs for thifensulfuron methyl. PBSA_2/4 is the MM-PBSA with ε of 2 or 4. The GB^OBC^ and GBn models are represented by 5 and 7, respectively. Binding affinity was computed on the basis of single structure minimized (SS), structures sampled from classical MD simulations (cMD), or structures extracted from QM/MM MD simulations (qMD).

For each *Ks*AHAS-herbicide complex, the binding affinity was estimated by 24 MM or QM/MM methods in combination with three different sets of structures obtained from structure minimization (i.e., single structure), classical MD simulations, and QM/MM MD simulations. The predictive power of different approaches to distinguish resistant mutants from susceptible ones was evaluated by the following three metrics: EF, AUC, and accuracy (see **S1–S4 Tables**). As shown in **Fig 4A and 4B**, the best prediction performance was achieved by MM-PBSA combined with single structure. With this approach, AUC and accuracy were both above 0.9 while EF reached the highest value of 1.16 for both herbicides. The internal dielectric constant (ε) had little influence on the results. When ε changed from 2 to 4, accuracy was slightly improved from 0.93 to 1, and AUC rose from 0.94 to 1 for tribenuron methyl. However, there was no change in EF, AUC, and accuracy for thifensulfuron methyl. The top three ROCs for tribenuron methyl and thifensulfuron methyl (see **S5 and S6 Tables**) were plotted and shown in **Fig 4C and 4D**, respectively. For thifensulfuron methyl, the ROCs of MM-PBSA with ε being either 2 or 4 were the same, so only the ROC of MM-PBSA with ε of 2 was presented (**Fig 4D**). Among QM/MM-GBSA methods, PM6D-GBn (for tribenuron methyl, **Fig 4C**) and AM1D-GB^OBC^ (for thifensulfuron methyl, **Fig 4D**) also demonstrated a high capability in differentiating between mutations causing resistance or susceptibility. Based on an ensemble of structures obtained from classical MD simulations, methods such as MM-PBSA, AM1D, and PM6D were better than most of the others. They were only inferior to the best performer (MM-PBSA combined with single structure). As AM1D performed well with both single structure and classical MD, it was chosen for depiction of the QM region in the QM/MM MD simulations. With an ensemble of structures from QM/MM MD, the QM/MM approaches showed similar discriminating ability for either herbicide.

## Discussion

It is noteworthy that there was a large variation in the discerning ability of the tested methods on the basis of single structure (**Fig 4A and 4B**). MM-PBSA combined with single structure was the frontrunner among all approaches, but some QM/MM GBSA methods with the same sampling strategy led to the worst performance in this work. By contrast, the discriminating power remained stable across different approaches based on an ensemble of structures from either classical or QM/MM MD simulations. It was also observed that the ability of QM/MM-GBSA to distinguish resistance mutations depended on the GB model and SQM correction. Here we further discuss how sampling techniques (i.e., single structure, classical MD, and QM/MM MD), GB models (GB^OBC^ and GBn), and SQM corrections (D and DH) affected identification of resistance mutations using different methods for binding affinity calculation.

**Fig 5:**
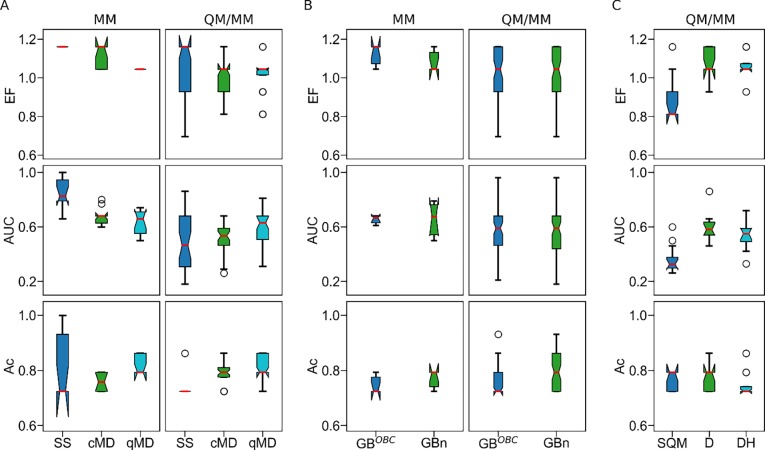
**Effects of sampling technique (A), GB model (B), and SQM corrections (C) on the performance metrics of MM and QM/MM-GBSA methods.** SQM correction impact was assessed for QM/MM-GBSA methods only. In each boxplot, red lines, notches, upper/lower whickers, upper/lower box border lines, and circles represent medians, confidence intervals of the medians, maximum/minimum values, 75/25 percentiles, and outliers, respectively.

The influence of sampling technique is summarized in **Fig 5A** and **Table 2**. For MM methods, single structure outperformed both classical MD and QM/MM MD in terms of EF and AUC, whereas the difference between classical MD and QM/MM MD was subtle. Specifically, the EF of classical MD was slightly better than that of QM/MM MD (*p*-value < 0.001), but QM/MM MD had a higher accuracy than classical MD (*p*-value < 0.01). For QM/MM-GBSA methods, QM/MM MD was significantly superior to single structure in terms of AUC (*p*-value < 0.01) and accuracy (*p*-value < 0.001) and was also better than classical MD in terms of EF (*p*-value < 0.05) and AUC (*p*-value < 0.001). The statistical difference between single structure and classical MD cannot be confirmed because the EF of single structure was statistically higher than that of classical MD (*p*-value < 0.05), but the accuracy of classical MD was better than that of single structure (*p*-value < 0.001). These results suggest that, when MM methods were employed to compute binding affinity, single structure was more suitable for distinguishing resistance mutations than the ensemble of structures from MD simulations. With QM/MM-GBSA methods in use, the ensemble of structures sampled from QM/MM MD simulations was preferred.

**Table 2:** Impact of sampling technique on the discerning ability of binding affinity estimation approaches. SS: single structure; cMD: classical MD; qMD: QM/MM MD.

**Calculation** **method**	**Sampling** **technique**	**Mean ± standard deviation**			***p*-value**					
		EF	AUC	Accuracy	EF		AUC		Accuracy	
					cMD	qMD	cMD	qMD	cMD	qMD
**MM**	SS	1.16±0.00	0.84±0.11	0.80±0.11	0.038	<0.001	<0.001	<0.001	0.152	0.599
	cMD	1.12±0.05	0.67±0.06	0.76±0.03		<0.001		0.387		0.002
	qMD	1.04±0.00	0.64±0.09	0.82±0.03						
**QM/MM-GBSA**	SS	1.04±0.15	0.49±0.19	0.73±0.02	0.045	0.539	0.543	0.009	<0.001	<0.001
	cMD	0.98±0.10	0.51±0.12	0.79±0.05		0.026		<0.001		0.057
	qMD	1.02±0.10	0.59±0.13	0.80±0.04						

The above results are in agreement with a previous study which reported that single structure performed as well as or better than MD simulations [60]. However, it is well known that, with single structure, the results depend on the starting structure, given that conformational changes are ignored. In this work, herbicides were first docked into the *At*AHAS, and the resulting structures were used as templates for building the *Ks*AHAS-herbicide complexes. In a preliminary study, we tested an alternative modeling strategy, in which the *Ks*AHAS structures (WT and mutated) were modeled first before the herbicides were docked into them. We observed that its discriminating power was unsatisfactory and much worse than what is reported here. Combined with single structure, most of the QM/MM-GBSA approaches were inferior to the MM approaches. This was possibly because the starting structures were energy-minimized using the MM force field and only one structure was adopted in the calculation of binding affinity. MM-PBSA performed better than QM/MM-GBSA possibly because the PB model was more accurate and less computationally expensive than the GB model. The lower accuracy of QM/MM-GBSA was likely introduced by the GB model. Nevertheless, more in-depth research is warranted to answer such questions as why certain methods performed better than others on a specific ligand-protein system.

In binding affinity calculation, the solvation effect is usually estimated by implicit solvent models [60]. Between the two implicit solvent models used for calculation of binding affinity in this study, there was no statistical difference for MM methods in terms of EF, AUC, and accuracy (*p*-values > 0.05, see **Fig 5B** and **Table 3**). For QM/MM-GBSA methods, the GBn model achieved a significantly greater accuracy (*p*-value < 0.001) than the GB^OBC^ model. Generally speaking, the GBn model was a better choice than the GB^OBC^ model for identification of resistance mutations, which worked well for both MM and QM/MM-GBSA methods.

**Table 3:** Impact of GB (generalized Born) model on the discriminating ability of binding affinity estimation methods.

**Calculation method**	**GB model**	**Mean ± standard deviation**			*p***-value**		
		EF	AUC	Accuracy	EF	AUC	Accuracy
MM	GB^OBC^	1.12±0.05	0.65±0.03	0.75±0.03	0.175	0.971	0.175
	GBn	1.08±0.05	0.66±0.12	0.77±0.10			
QM/MM-GBSA	GB^OBC^	1.03±0.11	0.56±0.16	0.77±0.05	0.694	0.885	<0.001
	GBn	1.02±0.12	0.56±0.17	0.79±0.06			

For the two SQM methods (AM1 and PM6), dispersion (D) and hydrogen bond (H) corrections were taken into account in this work. Compared to methods without corrections (i.e., AM1 and PM6), the SQMs with corrections (D and DH) notably enhanced prediction power measured by EF and AUC (*p*-values < 0.001, see **Fig 5C** and **Table 4**), which is consistent with previous studies [28, 29]. However, the addition of hydrogen bond correction (AM1DH and PM6DH) resulted in lower AUC than the dispersion correction only (AM1D and PM6D, *p*-value < 0.05), even though there was no statistical difference in EF and accuracy between them (*p*-values > 0.05). Therefore, SQM corrections were able to ameliorate the prediction performance of QM/MM-GBSA methods, and dispersion correction was more important than hydrogen bond correction.

**Table 4:** Impact of SQM (semi-empirical quantum mechanics) corrections on the discerning ability of QM/MM-GMSA methods.

SQM correction	Mean ± standard deviation			*p*-value					
	EF	AUC	Accuracy	EF		AUC		Accuracy	
				D	DH	D	DH	D	DH
**SQM**	0.89±0.11	0.36±0.10	0.77±0.03	<0.001	<0.001	<0.001	<0.001	1.0	0.104
**D**	1.07±0.07	0.59±0.10	0.77±0.04		0.166		0.019		0.166
**DH**	1.05±0.07	0.53±0.10	0.75±0.04						

The structural and functional characterization of the interaction between herbicides and their biomacromolecular targets is critical for better understanding and accurately assessing genetic mutation-conferred resistance. Therefore, modeling of *Ks*AHAS-herbicide complexes is an important aspect of recognizing resistance mutations. It is impractical to acquire experimental data to exhaustively examine all variables such as plant species, mutation, and herbicide due to inhibitory costs. As a result, many herbicide-enzyme complexes are not well characterized. To safeguard the effectiveness and sustainability of herbicide-based invasive plant management, it is vital to accurately assess the impact of mutations on ligand binding. In this work, we modeled the complex structures of a WT and 28 mutant *Ks*AHAS bound with two sulfonylurea herbicides (tribenuron methyl and thifensulfuron methyl), and investigated the ability of computational methods to distinguish between mutations causing resistance or susceptibility to either herbicide. Up to 100% accuracy was achieved when MM-PBSA combined with minimized single structure was employed to estimate the binding affinity of the two herbicides to the 29 *Ks*AHAS structures. Although we observed that all susceptible mutants had mutations outside the binding pocket while the resistant mutants possessed mutations inside the binding pocket, it is premature to extrapolate this observation to other mutations due to both the small sample size in this study and the high number of possible single-point mutations and combinations of multi-point mutations.

The *Ks*AHAS-herbicide sensitivity dataset used in this study was imbalanced with more resistant biotypes than susceptible ones. Imbalanced datasets may present a challenge to machine learning algorithms when the minority class is of interest, because these algorithms train a model by learning the features of the entire dataset. The more members a class has, the more the class is represented in the model. Although the imbalanced *K*. *scoparia* dataset was not an ideal one, it served the objective of this study, i.e., compare a wide variety of biophysics-based *in silico* methods and identify the best one for discerning mutation-conferred herbicide resistance in field populations of invasive plant species. Here, we compared 24 different MM and QM/MM methods combined with three different structure sampling strategies (**Fig 4** and **S1–S4 Tables**). These methods showed differential performance, and MM-PBSA with single structure was identified as the best approach for the specific system of AHAS-herbicide complexes.

To further evaluate the impact of dataset imbalance on method performance, we conducted a sensitivity test by randomly selecting 4 to 24 resistant mutants together with all 3 sensitive mutants and the wild-type *Ks*AHAS, and recalculating prediction accuracy for the new datasets. We plotted the mean and standard deviation of accuracy against the number of selected resistant mutants for seven scenarios of single structure sampling: MM-PBSA (*ε* = 2), MM-PBSA (*ε* = 4) and QM/MM-GBSA for both tribenuron methyl and thifensulfuron methyl, and MM-GBSA for thifensulfuron methyl (**S1 Fig**). The accuracy of 1000 unique random combinations (except for 25 combinations of 24 resistant mutants and 300 combinations of 23 resistant mutants, both of which were the maximum number of all possible non-redundant combinations) was influenced by the degree of dataset imbalance (i.e., ratio of resistant to sensitive mutants) in six of the seven scenarios. While the mean accuracy was little affected, variation in accuracy increased as the dataset became more balanced (i.e., fewer resistant mutants included). Obviously, MM-PBSA had smaller variations in accuracy and was more resistant to dataset imbalance than MM-GBSA and QM/MM-GBSA. Especially, the MM-PBSA method with *ε* = 4 for tribenuron methyl was not affected by imbalance because it determined that all resistant *Ks*AHAS mutants had a lower binding affinity with the herbicide than the wild-type and sensitive mutants.

In summary, we present here how homology modeling, docking, and MD simulations were integrated to computationally predict the resistance of a mutated enzyme to an herbicide with no prior knowledge of the complex structure. The estimation of noncovalent interaction remains a big challenge in accurate identification of resistance mutations because binding affinity calculation is computationally expensive, and the calculation accuracy depends on sampling techniques and estimation methods. However, with increasing computational power, noncovalent interactions can be precisely modeled with more rigorous methods, and the complex structure can be sampled more thoroughly. This study demonstrates that excellent agreement between *in silico* prediction and experimental data of herbicide resistance can be achieved when appropriate computational approaches are chosen.

## Disclaimer

The content is solely the responsibility of the authors and does not necessarily represent the official views of U.S. Army Corps of Engineers and U.S. Food and Drug Administration.

## Supporting information

S1 Fig Influence of dataset imbalance on prediction accuracy of select methods. A sensitivity test was performed to examine the impact of dataset imbalance (i.e., the ratio between resistant (R) to sensitive (S) mutants) on method performance (evaluated using accuracy). See S5 and S6 Tables for the data input. The number of resistant mutants varied from 4 (R:S = 1:1) to 24 (R:S = 6:1). Shown are the mean ± standard deviation (n = 1000 unique random combinations, except 25 and 300 combinations of 24 and 23 resistant mutants included, respectively, because they were the maximum number of all possible non-redundant combinations).
(DOCX)

S1 Table Enrichment factor (EF), area under the ROC curve (AUC), and accuracy of MM-PBSA models based on single structure (SS), classical MD (cMD), and QM/MM MD simulations (qMD) for two AHAS-inhibiting herbicides, tribenuron methyl (TBM) and thifensulfuron methyl (TFM). (DOCX)

S2 Table Enrichment factor (EF), area under the ROC curve (AUC), and accuracy of MM-GBSA/ALPB and QM/MM-GBSA models based on single structure for two AHAS-inhibiting herbicides, tribenuron methyl (TBM) and thifensulfuron methyl (TFM). (DOCX)

S3 Table Enrichment factor (EF), area under the ROC curve (AUC), and accuracy of MM-GBSA/ALPB and QM/MM-GBSA based on an ensemble of structures sampled from classical MD simulations for two AHAS-inhibiting herbicides, tribenuron methyl (TBM) and thifensulfuron methyl (TFM). (DOCX)

S4 Table Enrichment factor (EF), area under the ROC curve (AUC), and accuracy of MM-GBSA/ALPB and QM/MM-GBSA based on an ensemble of structures sampled from QM/MM MD simulations for two AHAS-inhibiting herbicides, tribenuron methyl (TBM) and thifensulfuron methyl (TFM). (DOCX)

S5 Table Estimated binding affinity of tribenuron methyl with *Ks*AHASs. Single structure was used for all methods. The *Ks*AHASs were sorted in descending order of estimated binding affinity.
(DOCX)

S6 Table Estimated binding affinity of thifensulfuron methyl with *Ks*AHASs. Single structure was used for all methods. The *Ks*AHASs were sorted in descending order of estimated binding affinity.
(DOCX)
