# Supplementary material for: In silico identification of genetic mutations conferring resistance to acetohydroxyacid synthase inhibitors: A case study of Kochia scoparia
Source: PLoS One. 2019 May 7;14(5):e0216116. doi: 10.1371/journal.pone.0216116 (PMC6504096; doi:10.1371/journal.pone.0216116)
Supplement: S1 Fig — A sensitivity test was performed to examine the impact of dataset imbalance (i.e., the ratio between resistant (R) to sensitive (S) mutants) on method performance (evaluated using accuracy). See S5 and S6 Tables for the data input. The number of resistant mutants varied from 4 (R:S = 1:1) to 24 (R:S = 6:1). Shown are the mean ± standard deviation (n = 1000 unique random combinations, except 25 and 300 combinations of 24 and 23 resistant mutants included, respectively, because they were the maximum number of all possible non-redundant combinations). (DOCX) [file pone.0216116.s001.docx]

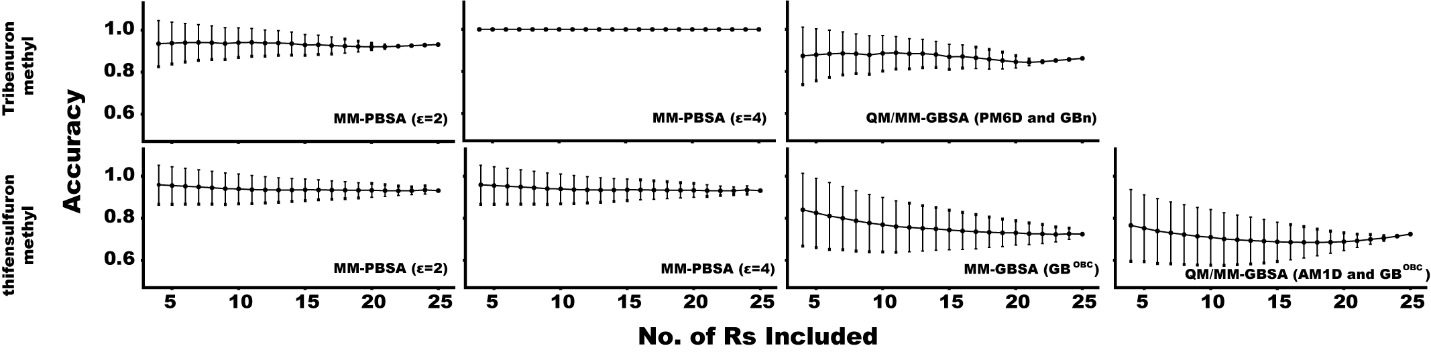


**S1 Fig.** **Influence of dataset imbalance on prediction accuracy of select methods.**

A sensitivity test was performed to examine the impact of dataset imbalance (i.e., the ratio between resistant (R) to sensitive (S) mutants) on method performance (evaluated using accuracy). See Tables S5 and S6 for the data input. The number of resistant mutants varied from 4 (R:S = 1:1) to 24 (R:S = 6:1). Shown are the mean ± standard deviation (n = 1000 unique random combinations, except 25 and 300 combinations of 24 and 23 resistant mutants included, respectively, because they were the maximum number of all possible non-redundant combinations).
