# Supplementary material for: In silico identification of genetic mutations conferring resistance to acetohydroxyacid synthase inhibitors: A case study of Kochia scoparia
Source: PLoS One. 2019 May 7;14(5):e0216116. doi: 10.1371/journal.pone.0216116 (PMC6504096; doi:10.1371/journal.pone.0216116)
Supplement: S1 Table — (DOCX) [file pone.0216116.s002.docx]

**S1 Table.** **Enrichment factor (EF), area under the ROC curve (AUC), and accuracy of MM-PBSA models based on single structure (SS), classical MD (cMD), and QM/MM MD simulations (qMD) for two AHAS-inhibiting herbicides, tribenuron methyl (TBM) and thifensulfuron methyl (TFM).**

| **Sampling** | **Herbicide** | $\boldsymbol{\varepsilon=2}$ | | | $\boldsymbol{\varepsilon=4}$ | | |
| --- | --- | --- | --- | --- | --- | --- | --- |
|  |  | **EF** | **AUC** | **Accuracy** | **EF** | **AUC** | **Accuracy** |
| **SS** | **TBM** | 1.16 | 0.94 | 0.93 | 1.16 | 1 | 1 |
|  | **TFM** | 1.16 | 0.96 | 0.93 | 1.16 | 0.96 | 0.93 |
| **cMD** | **TBM** | 1.16 | 0.77 | 0.79 | 1.16 | 0.80 | 0.79 |
|  | **TFM** | 1.04 | 0.63 | 0.79 | 1.04 | 0.62 | 0.79 |
| **qMD** | **TBM** | 1.04 | 0.56 | 0.79 | 1.04 | 0.53 | 0.79 |
|  | **TFM** | 1.04 | 0.74 | 0.86 | 1.04 | 0.70 | 0.86 |
