# Supplementary material for: In silico identification of genetic mutations conferring resistance to acetohydroxyacid synthase inhibitors: A case study of Kochia scoparia
Source: PLoS One. 2019 May 7;14(5):e0216116. doi: 10.1371/journal.pone.0216116 (PMC6504096; doi:10.1371/journal.pone.0216116)
Supplement: S3 Table — (DOCX) [file pone.0216116.s004.docx]

**S3 Table. Enrichment factor (EF), area under the ROC curve (AUC), and accuracy of MM-GBSA/ALPB and QM/MM-GBSA based on an ensemble of structures sampled from classical MD simulations for two AHAS-inhibiting herbicides, tribenuron methyl (TBM) and thifensulfuron methyl (TFM).**

| **Herbicide** | **Method** | | **GB^OBC^** | | | **GBn** | | |
| --- | --- | --- | --- | --- | --- | --- | --- | --- |
|  |  |  | **EF** | **AUC** | **Accuracy** | **EF** | **AUC** | **Accuracy** |
| **TBM** | **MM** | GBSA | 1.16 | 0.68 | 0.72 | 1.04 | 0.67 | 0.79 |
|  |  | ALPB | 1.16 | 0.68 | 0.72 | 1.04 | 0.68 | 0.79 |
|  | **QM/MM-GBSA** | PM3 | 0.93 | 0.33 | 0.79 | 0.81 | 0.30 | 0.79 |
|  |  | RM1 | 0.93 | 0.33 | 0.79 | 0.81 | 0.29 | 0.79 |
|  |  | AM1 | 0.81 | 0.31 | 0.79 | 0.81 | 0.26 | 0.79 |
|  |  | AM1D | 1.16 | 0.66 | 0.72 | 1.04 | 0.55 | 0.79 |
|  |  | AM1DH | 1.16 | 0.61 | 0.72 | 1.04 | 0.47 | 0.72 |
|  |  | PM6 | 0.81 | 0.30 | 0.79 | 0.93 | 0.34 | 0.79 |
|  |  | PM6D | 1.04 | 0.59 | 0.79 | 0.93 | 0.50 | 0.79 |
|  |  | PM6DH | 1.04 | 0.58 | 0.72 | 0.93 | 0.50 | 0.86 |
|  |  | DFTB3 | 0.93 | 0.52 | 0.79 | 0.93 | 0.45 | 0.79 |
| **TFM** | **MM** | GBSA | 1.16 | 0.60 | 0.72 | 1.16 | 0.68 | 0.72 |
|  |  | ALPB | 1.16 | 0.60 | 0.72 | 1.16 | 0.68 | 0.72 |
|  | **QM/MM-GBSA** | PM3 | 0.93 | 0.54 | 0.86 | 0.93 | 0.53 | 0.86 |
|  |  | RM1 | 1.04 | 0.54 | 0.79 | 0.93 | 0.52 | 0.86 |
|  |  | AM1 | 1.04 | 0.53 | 0.79 | 0.93 | 0.53 | 0.86 |
|  |  | AM1D | 1.04 | 0.55 | 0.72 | 1.04 | 0.63 | 0.86 |
|  |  | AM1DH | 1.04 | 0.49 | 0.72 | 1.04 | 0.59 | 0.72 |
|  |  | PM6 | 1.04 | 0.60 | 0.79 | 0.93 | 0.59 | 0.86 |
|  |  | PM6D | 1.04 | 0.59 | 0.72 | 1.04 | 0.67 | 0.86 |
|  |  | PM6DH | 1.04 | 0.55 | 0.72 | 1.04 | 0.68 | 0.79 |
|  |  | DFTB3 | 1.16 | 0.67 | 0.79 | 1.04 | 0.65 | 0.86 |
