# Supplementary material for: In silico identification of genetic mutations conferring resistance to acetohydroxyacid synthase inhibitors: A case study of Kochia scoparia
Source: PLoS One. 2019 May 7;14(5):e0216116. doi: 10.1371/journal.pone.0216116 (PMC6504096; doi:10.1371/journal.pone.0216116)
Supplement: S5 Table — Single structure was used for all methods. The KsAHASs were sorted in descending order of estimated binding affinity. (DOCX) [file pone.0216116.s006.docx]

**S5 Table. Estimated binding affinity of tribenuron methyl with *Ks*AHASs**. Single structure was used for all methods. The *Ks*AHASs were sorted in descending order of estimated binding affinity.

| **MM-PBSA (ε=2)** | | | **MM-PBSA (ε=4)** | | | **QM/MM-GBSA (PM6D and GBn)** | | |
| --- | --- | --- | --- | --- | --- | --- | --- | --- |
| ***Ks*AHAS** | **BA**^a^ | **A^b^** | ***Ks*AHAS** | **BA** | **A** | ***Ks*AHAS** | **BA** | **A** |
| P197Q+W574R | -4.38 | R^c^ | P197Q+W574R | -11.02 | R | P197K | -3.34 | R |
| W574R | -7.00 | R | W574R | -13.01 | R | P197S+D376E | -3.36 | R |
| P197W | -7.96 | R | P197W | -13.88 | R | P197Q+W574R | -3.36 | R |
| P197T+W574L | -9.28 | R | P197T+W574L | -14.58 | R | D376E+W574L | -3.83 | R |
| P197Q+W574L | -9.80 | R | P197A+W574L | -15.07 | R | P197A+W574L | -3.86 | R |
| P197K | -10.08 | R | P197Q+W574L | -15.07 | R | W574R | -4.11 | R |
| P197S+W574L | -10.29 | R | P197S+W574L | -15.11 | R | P197A | -4.52 | R |
| P197A+W574L | -10.43 | R | P197S+D376E | -16.14 | R | P197S+W574L | -4.89 | R |
| P197T | -10.80 | R | P197T | -16.24 | R | P197R+W574L | -5.38 | R |
| P197E | -11.02 | R | P197K | -16.47 | R | D376E | -5.69 | R |
| P197Q | -11.09 | R | P197A | -16.68 | R | P197Q+D376E | -5.73 | R |
| P197L+W574L | -11.37 | R | P197E | -16.71 | R | P197Q+W574L | -5.78 | R |
| P197A | -11.46 | R | P197Q | -16.78 | R | P197R | -5.86 | R |
| P197S+D376E | -11.92 | R | P197L+W574L | -16.80 | R | P197L+W574L | -6.08 | R |
| P197S | -12.48 | R | P197S | -17.32 | R | P197T+D376E | -6.75 | R |
| P197L | -12.62 | R | P197Q+D376E | -17.32 | R | W574L | -6.77 | R |
| P197M | -12.86 | R | P197T+D376E | -17.39 | R | P197T+W574L | -6.87 | R |
| P197Q+D376E | -13.04 | R | W574L | -17.41 | R | P197E | -7.00 | R |
| W574L | -13.05 | R | D376E+W574L | -17.47 | R | P197L | -7.15 | R |
| P197R+W574L | -13.46 | R | P197R+W574L | -17.59 | R | E284V | -7.75 | S |
| P197T+D376E | -13.59 | R | P197M | -18.41 | R | V225I | -7.89 | R |
| V225I | -14.06 | R | P197L | -18.48 | R | P197M | -7.99 | R |
| E284V | -14.07 | S^d^ | D376E | -18.88 | R | G268D | -8.05 | S |
| D376E+W574L | -14.23 | R | P197R | -19.08 | R | P197Q | -8.15 | R |
| P197R | -14.31 | R | V225I | -19.11 | R | P197T | -8.15 | R |
| G268D | -14.33 | S | E284V | -19.16 | S | WT | -8.30 | S |
| N434K | -14.60 | S | G268D | -19.31 | S | N434K | -8.64 | S |
| WT | -14.76 | S | N434K | -19.45 | S | P197S | -8.92 | R |
| D376E | -15.09 | R | WT | -19.45 | S | P197W | -9.12 | R |

^a^BA: binding affinity (kcal/mol); ^b^A: experimentally determined activity; ^c^R: resistant; ^d^S: susceptible
