# Supplementary material for: In silico identification of genetic mutations conferring resistance to acetohydroxyacid synthase inhibitors: A case study of Kochia scoparia
Source: PLoS One. 2019 May 7;14(5):e0216116. doi: 10.1371/journal.pone.0216116 (PMC6504096; doi:10.1371/journal.pone.0216116)
Supplement: S6 Table — Single structure was used for all methods. The KsAHASs were sorted in descending order of estimated binding affinity. (DOCX) [file pone.0216116.s007.docx]

**S6 Table. Estimated binding affinity of thifensulfuron methyl with *Ks*AHASs.** Single structure was used for all methods. The *Ks*AHASs were sorted in descending order of estimated binding affinity.

| **MM-PBSA (ε=2)** | | | **MM-PBSA (ε=4)** | | | **MM-GBSA (GB^OBC^)** | | | **QM/MM-GBSA (AM1D**  **and GB^OBC^)** | | |
| --- | --- | --- | --- | --- | --- | --- | --- | --- | --- | --- | --- |
| ***Ks*AHAS** | **BA**^a^ | **A^b^** | ***Ks*AHAS** | **BA** | **A** | **AHAS** | **BA** | **A** | ***Ks*AHAS** | **BA** | **A** |
| P197Q+W574R | 3.74 | R^c^ | P197Q+W574R | -5.26 | R | P197T+W574L | -31.00 | R | P197E | -11.42 | R |
| W574R | 2.42 | R | P197T+W574L | -5.89 | R | P197S+W574L | -31.56 | R | P197T+W574L | -12.45 | R |
| P197T+W574L | 0.70 | R | P197L+W574L | -6.28 | R | P197A+W574L | -31.57 | R | P197L+W574L | -14.17 | R |
| P197L+W574L | 0.60 | R | P197S+W574L | -6.31 | R | P197L+W574L | -32.27 | R | P197S+W574L | -14.56 | R |
| P197S+W574L | -0.29 | R | P197A+W574L | -6.82 | R | W574R | -32.75 | R | P197A+W574L | -14.76 | R |
| P197Q+W574L | -0.83 | R | P197Q+W574L | -7.09 | R | P197Q+W574R | -32.79 | R | P197W | -14.85 | R |
| P197A+W574L | -0.93 | R | W574R | -7.16 | R | P197R+W574L | -33.30 | R | P197T | -15.55 | R |
| P197R+W574L | -2.77 | R | P197R+W574L | -8.44 | R | W574L | -34.23 | R | W574L | -15.98 | R |
| D376E+W574L | -2.85 | R | D376E+W574L | -8.59 | R | P197Q+W574L | -34.39 | R | P197Q+W574R | -16.34 | R |
| P197S+D376E | -3.32 | R | P197E | -9.88 | R | D376E+W574L | -35.13 | R | W574R | -16.35 | R |
| P197T | -3.35 | R | W574L | -10.02 | R | P197E | -35.32 | R | P197R+W574L | -16.77 | R |
| P197T+D376E | -3.59 | R | P197S+D376E | -10.22 | R | P197T | -35.96 | R | P197Q+W574L | -16.94 | R |
| P197S | -4.01 | R | P197T | -10.27 | R | P197A | -36.62 | R | P197A | -17.33 | R |
| P197E | -4.03 | R | P197S | -10.55 | R | P197T+D376E | -36.71 | R | D376E+W574L | -17.55 | R |
| P197L | -4.28 | R | P197T+D376E | -10.71 | R | P197S | -36.72 | R | P197S | -17.83 | R |
| P197A | -4.32 | R | P197A | -10.76 | R | P197S+D376E | -37.33 | R | P197L | -18.00 | R |
| P197K | -4.83 | R | P197L | -11.07 | R | P197L | -37.55 | R | P197T+D376E | -18.18 | R |
| W574L | -4.85 | R | P197Q | -11.74 | R | P197W | -38.44 | R | P197M | -18.29 | R |
| P197Q | -4.98 | R | P197Q+D376E | -12.04 | R | P197R | -38.76 | R | E284V | -19.31 | S |
| P197W | -5.59 | R | P197K | -12.16 | R | P197M | -39.00 | R | P197S+D376E | -19.35 | R |
| P197Q+D376E | -5.62 | R | P197W | -12.47 | R | P197K | -39.49 | R | V225I | -19.36 | R |
| P197R | -6.89 | R | P197R | -12.77 | R | G268D | -39.57 | S | WT | -19.55 | S |
| D376E | -7.37 | R | D376E | -13.85 | R | N434K | -39.61 | S | N434K | -19.77 | S |
| V225I | -8.22 | R | V225I | -14.07 | R | E284V | -39.67 | S | G268D | -19.85 | S |
| G268D | -8.35 | S^d^ | E284V | -14.14 | S | WT | -39.75 | S | P197R | -20.15 | R |
| WT | -8.36 | S | WT | -14.20 | S | V225I | -39.77 | R | P197Q | -20.36 | R |
| E284V | -8.37 | S | G268D | -14.28 | S | P197Q | -39.89 | R | P197K | -21.45 | R |
| N434K | -8.78 | S | N434K | -14.49 | S | D376E | -40.59 | R | D376E | -21.81 | R |
| P197M | -9.68 | R | P197M | -15.00 | R | P197Q+D376E | -41.16 | R | P197Q+D376E | -22.30 | R |

^a^BA: binding affinity (kcal/mol); ^b^A: experimentally determined activity; ^c^R: resistant; ^d^S: susceptible
